# Solvent Engineering for Intermediates Phase, All-Ambient-Air-Processed in Organic–Inorganic Hybrid Perovskite Solar Cells

**DOI:** 10.3390/nano9070915

**Published:** 2019-06-26

**Authors:** Lei Shi, Huiying Hao, Jingjing Dong, Tingting Zhong, Chen Zhang, Jiabin Hao, Jie Xing, Hao Liu

**Affiliations:** School of Science, China University of Geosciences, Beijing 100083, China

**Keywords:** perovskite solar cells, intermediate phase, air condition, solvent engineering

## Abstract

Intermediate phase is considered an important aspect to deeply understand the crystallization procedure in the growth of high-quality perovskite layers by an anti-solvent technique. However, the moisture influence on the intermediate phase formation is not clear in air conditions as yet. In this work, pure (FA_0.2_MA_1.8_)Pb_3_X_8_(DMSO·DMF) intermediate phase was obtained in as-prepared perovskite film by spin-coating the precursor of co-solvent (DMSO and DMF) in an ambient air (RH20–30%). Moreover, the appropriate quantity of ethyl acetate (C_4_H_8_O_2_, EA) also controls the formation of pure intermediate phase. The uniform and homogeneous perovskite film was obtained after annealing this intermediate film. Therefore, the best power conversion efficiency (PCE) of perovskite solar cells (PSCs) is 16.24% with an average PCE of 15.53%, of which almost 86% of its initial PCE was preserved after 30 days in air conditions. Besides, the steady-state output efficiency ups to 15.38% under continuous illumination. In addition, the PCE of large area device (100 mm^2^) reaches 11.11% with a little hysteresis effect. This work would give an orientation for PSCs production at the commercial level, which could lower the cost of fabricating the high efficiency PSCs.

## 1. Introduction

Organic–inorganic hybrid perovskite solar cells (PSCs) have revolutionized the field of photovoltaics, with rapid progress in power conversion efficiency (PCE) from 3.8% in 2009 [1] to the present record of 24.2% [2]. Great progress has been made for different device configurations, including the classic n–i–p structures and inverted p–i–n structures according to the sequence of thin film deposition [3]. Whatever the configuration is, the high performance of PSCs can be attributed to excellent photoelectric properties of perovskite layer such as high absorption, long carrier diffusion lengths, and appropriate band gap [4,5]. Various synthesis approaches to obtain high quality perovskite layer have been reported, including sequential deposition of inorganic and organic components, single-step spin-coating, thermal evaporation, and vapor-assisted method [6,7,8]. The single-step spin-coating method is generally used due to the ease of processing. To obtain the uniform and pinhole-free perovskite layer, anti-solvent treatment has been developed using solvents such as toluene and chlorobenzene during the spin-coating process [9,10,11]. The anti-solvent is miscible in the precursor solvents, but is not insoluble in the perovskite component. In this way, all precursor salts are dissolved in a mixed solvent, then spin-coated after the precursor solution is fully dissolved, with anti-solvent dropped on the top of the film during the spinning process. The precursor solution plays an important role in the excellent performance of PSCs. Generally, N,N-dimethylformamide (DMF), dimethylsulphoxide (DMSO) and γ-butyrolactone (GBL) or their mixture are used as solvents for precursor salts [12,13,14]. Jeon et al. suggested DMSO, and GBL (3:7 volume ratio) as a co-solvents for the precursors [15], which presented favorable surface coverage and smooth film with the toluene treatment, because the intermediate phase of CH_3_NH_3_I-PbI_2_-DMSO restricts the fast reaction between CH_3_NH_3_I and PbI_2_ during the rapid solvent evaporation in the spin-coating process. After that, Park et al. suggested combining the Lewis base DMSO, the iodide(I^-^) of CH_3_NH_3_I, and Lewis acid PbI_2_ to form an CH_3_NH_3_I-PbI_2_-DMSO intermediate phase via the adduct approach [16]. They demonstrated the formation of a 1:1:1 adduct of CH_3_NH_3_I-PbI_2_-DMSO film by spin-coating a DMF precursor solution containing equimolar CH_3_NH_3_I, PbI_2_, and DMSO, followed by anti-solvent diethyl ether drop-casting. In addition, Rong et al. used a mixed DMSO/DMF (3:1 volume ratio) co-solvent and toluene as the anti-solvent, identifying the intermediate phase structure as MA_2_Pb_3_I_8_(DMSO)_2_ [17]. Then, they suggested that this intermediate phase was a key point to form high-quality perovskite layer [18]. After that, Bai et al. obtained a pure MA_2_Pb_3_I_8_(DMSO)_2_ intermediate phase by regulating the ratio of DMSO:PbI_2_ in the perovskite precursor [19].

In order to maintain a superb performance of PSCs, the spin-coating process presented in the above works were all carried out under rigorous conditions in glove boxes with a small amount of oxygen and water, because the organic components in the perovskite structure is easily dissolved by moisture. This will undoubtedly increase the cost of manufacturing, and place restrictions on the eventual industrialization of PSCs. Therefore, fabricating perovskite films in ambient air is very important for technology’s commercialization. Due to the existence of moisture, the dynamical process of perovskite growth in ambient air might be quite different from that in glove boxes. Several issues on high quality perovskite fabrication in air need to be clarified, such as what the role of intermediate phase is, and what the effect of moisture, anti-solvent and precursor solution on the intermediate phase is. There are still quite few reports on this discussion. Besides, there is a lack of understanding of the intermediate phase contained both MA^+^ and FA^+^ although perovskite of mixed cation is widely used for compatible high efficiency and durability of PSCs.

Here, the effects of precursor solution, anti-solvent, and moisture on the formation of intermediate phase were investigated and the mechanism was discussed in air condition. By simply mixing solvent (DMSO/DMF = 5:0, 4:1, 3:2, 2:3) volume ratio of precursor solution, the (FA_0.2_MA_1.8_)Pb_3_X_8_(DMSO)_2_/perovskite phase and (FA_0.2_MA_1.8_)Pb_3_X_8_(DMSO·DMF) intermediate phase were acquired. In addition, the proper quantity of ethyl acetate (C_4_H_8_O_2_ EA) is also a key to obtain pure intermediate phase. Therefore, the best PCE of a device is 16.24%, and an average PCE is 15.53% by annealing pure (FA_0.2_MA_1.8_)Pb_3_X_8_(DMSO·DMF) intermediate film. The steady-state efficiency of the device is 15.38% under continuous illumination, almost 86% of its initial PCE was preserved after 30 days in air conditions. At the same humidity, an active area of 100 mm^2^ device was fabricated with a PCE of 11.11% and showed a small hysteresis between the reverse and forward. All the process of spin-coating was done in the ambient air (RH20–30%).

## 2. Materials and Methods

### 2.1. Materials

Materials used in our work include titanium (IV) isopropoxide (99.999%, Alfa Aesar, Shanghai, China) and PbI_2_ (99% Sigma-Aldrich, St. Louis, MO, USA). CH_3_NH_3_I (MAI, 99.5%, 4 times purification), CH_3_NH_3_Cl (MACl, 99.5%, 4 times purification), CH_3_(NH_2_)_2_I (FAI, 99.5%, 4 times purification), 2,2’,7,7’-Tetrakis[N,N-di(4-methoxyphenyl)amino]-9,9’-spirobifluorene (Spiro-OMeTAD, 99.5%), TiO_2_ paste (Dyesol-30NRD), Lithium bis(trifluoromethanesulfonyl)imide (Li-TFSI, 99%),Tris(2-(1H-pyrazol-1-yl)-4-tert-butylpyridine)-cobalt(III)Tris(bis(trifluoromethylsulfonyl) imide)) (FK209, 99%), and 4-tertbutylpyridine (TBP, 96%) were obtained from Xi’an Polymer Light Technology Crop. (Xi’an, China). The solvent and anti-solvent, including N,N-Dimethylformamide (DMF, 99.5%), Dimethyl sulfoxide (DMSO, 99.8%), methyl acetate (MA, 99.95%), ethyl acetate (EA, 99.95%) and chlorobenzene (CB, 99.95%) were obtained from Aladdin corporation (Shanghai, China).

### 2.2. Device Fabrication

The device of PSCs was fabricated with the following structure: FTO/C-TiO_2_/M-TiO_2_/perovskite/Spiro-OMeTAD/Ag. FTO glass substrates were cleaned in acetone, isopropanol, ethanol, and deionized water for 15 min by ultrasonic cleaner and then the clean substrates were dried in air. Plasma cleaning was further done to treat the substrate at the 40 W for 30 s. The 8 µL concentrated hydrochloric acid and 40 µL titanium(IV) isopropoxide were added in 1 mL ethanol to synthetize compact TiO_2_ (C-TiO_2_) precursor solution, then spin-coated at 3000 rpm for 30 s on FTO substrates and immediately annealed at 150 °C for 15 min on a hot plate. The same process was repeated twice and annealed at 500 °C for 30 min to obtain C-TiO_2_ layer. After that, the coated substrates were immersed into a 40 mM TiCl_4_ aqueous solution at 70 °C for 30 min and heat-treated at 500 °C for 30 min. Mesoporous TiO_2_ (M-TiO_2_) precursor was prepared by blending TiO_2_ paste with ethanol (weight ratio = 1:4). After stirring for 12 h at room temperature, it was then spin-coated at 5000 rpm for 45 s followed by annealing at 80 °C for 40 min and sintered at 500 °C for 30 min. 1.3 M perovskite precursor solution was prepared by mixing of PbI_2_, MAI, FAI, and MACl (molar ratio = 1:0.8:0.1:0.1) in co-solvent (V_DMSO:_V_DMF_ = 5:0, 4:1, 2:3, 3:2). Then, perovskite solutions were successively spin-coated on the substrates at 1000 rpm for 10 s and 5000 rpm for 30 s. 100 µL of ethyl acetate was dropped in 10 s at 5000 rpm and was annealed at 100 °C for 40 min. The 91 mg of Spiro-OMeTAD with additives were dissolved in 1 mL chlorobenzene to prepare hole transport layer (HTL) solution. The additives include 21 µL of Li-TFSI (520 mg in 1 mL of acetonitrile), 16 µL of FK209 (375 mg in 1 mL of acetonitrile), and 36 µL of TBP. The HTL was prepared by spin-coating the solution at 4000 rpm for 20s on perovskite film. Finally, there was the deposition of the 100 nm thick Ag electrode by thermal evaporation in a high vacuum (5 × 10^4^ Pa). All the processes of spin-coating was done in ambient air (RH20–30%).

### 2.3. Film and Device Characterization

J–V curves of the PSCs were taken using a Keithley 2400 source measure unit under simulated solar illumination of 100 mW cm^−2^ (AM 1.5G) in the air. The reverse scan is from 1.4–0.2 V and forward scan is from −0.2 to 1.4 V with scan rate of 100 mV/s. The FTIR spectra were recorded on Excalibur 3100 (varian, Palo Alto, CA, USA). X-ray diffraction (XRD) patterns were tested by X-ray diffractometer (Bruker, Karlsruhe, Germany) with Cu ka radiation. The surface and cross section morphological of the perovskite films and PSCs devices were recorded by the scanning electron microscope (SEM S-4800, HITACHI, Tokyo, Japan). Atomic force microscopy (AFM) of perovskite film was tested by the equipment of Bruker Dimension Icon. UV–vis absorption spectra of samples were measured by spectrophotometer (Cary 5000, Palo Alto, CA, USA). Steady-state photo-luminescence (PL) spectra and time resolved photoluminescence (TRPL) decay was measured by PL spectrometer (F900, Xianggan, Beijing, China) with an excitation wavelength of 510 nm and 375 nm respectively. The IPCE measurements were carried out by Zolix SCS10-X150-DZ system (Zolix, Beijing, China) with the DC mode.

## 3. Results and Discussion

**Figure 1:**
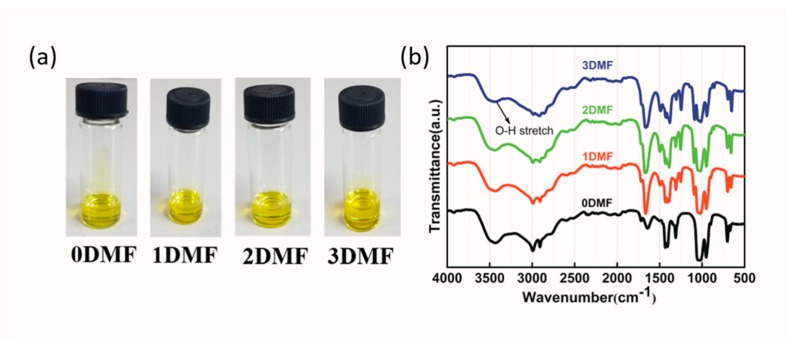
(**a**) Different perovskite precursor solution photos; (**b**) Fourier Transform Infrared (FTIR) transmittance spectra of precursor solutions.

The PbI_2_, MAI, FAI, and MACl were dissolved in co-solvent (V_DMSO_/V_DMF_ = 5:0, 4:1, 3:2, 2:3). The names of these precursor solutions correspond to 0DMF, 1DMF, 2DMF, and 3DMF, respectively. Figure 1a shows the perovskite precursor solutions of different solvent volume ratio. The solutions color was transparent yellow which indicated the solutes had been completely dissolved after 6 h stirring. Figure 1b shows Fourier Transform Infrared (FTIR) transmittance spectra of different precursors. The spectra exhibited apparent shallowing of the peak at 3435 cm^−1^ with increasing DMF content, corresponding to the O-H bond within the water molecule. That suggests absorption water in the solution, and as the amount of DMSO decreases, water is also decreasing, which is due to hydration of DMSO.

**Figure 2:**
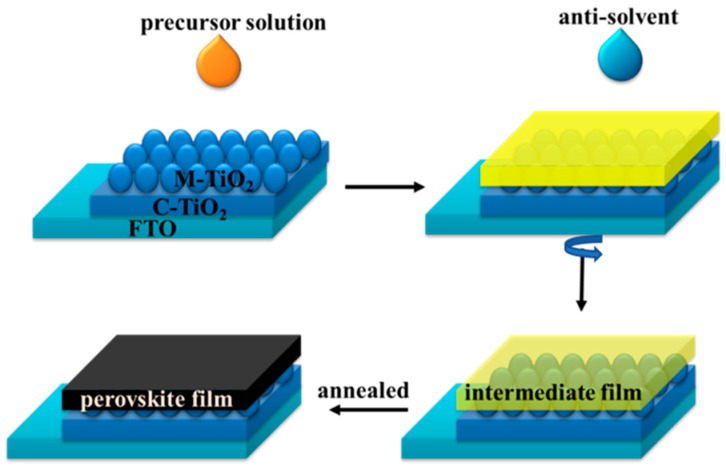
Schematic illustration of the perovskite film fabrication.

Figure 2 shows the schematic fabrication of perovskite film. The prepared precursor was spin-coated on mesoporous TiO_2_ (M-TiO_2_) by a one-step anti-solvent spin-coating processes at 1000 rpm for 10 s and 5000 rpm for 30 s successively [20]. During the spin-coating, EA was dripped on the spinning substrate in order to promote the formation of the intermediate phase [21]. In addition, EA was proved to avoid the air moisture into the intermediate phase [22,23]. Then, the intermediate film was transformed to pervoskite film after annealing at 100 °C for 40 min.

**Figure 3:**
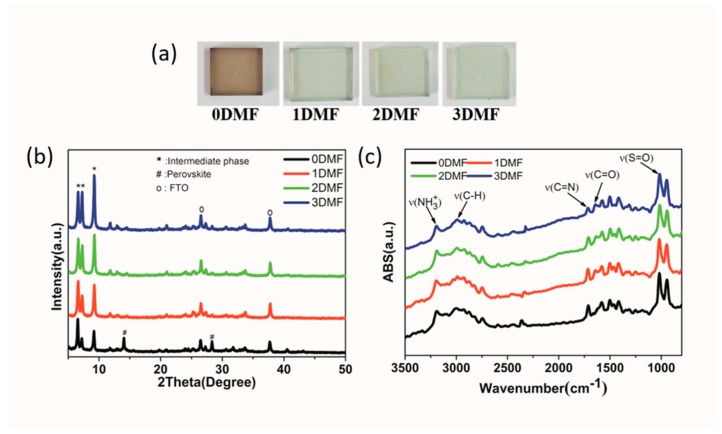
(**a**) The intermediate film photos of different precursors; (**b**) the X-ray diffraction (XRD) patterns and (**c**) Attenuated Total Reflectance Fourier Transform Infrared (ATR-FTIR) spectra of intermediate films.

**Figure 4:**
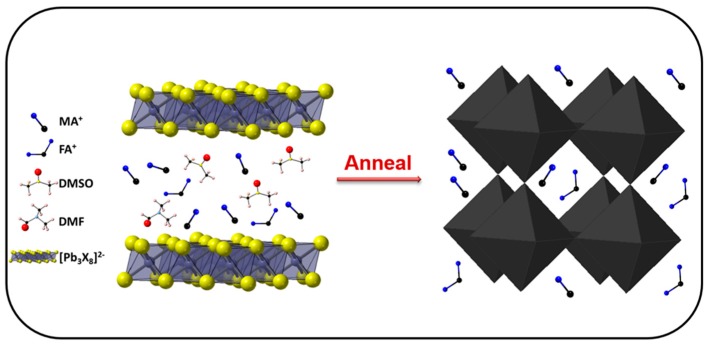
Schematic illustration of the intermediate phase (**left**) and perovskite phase (**right**).

The intermediate film (Figure 3a) exhibited a clear difference in color. The color of film is light brown when fabricated from pure DMSO solution (0DMF case). As DMF was added in the precursors, the color of films changed from light brown to transparent, that indicated the different composition of intermediate films. Figure 3b shows X-ray diffraction (XRD) patterns of different intermediate films deposited on FTO glass. The films showed identical peak positions compared to those previously reported of MA_2_Pb_3_I_8_(DMSO)_2_ intermediate phase [17]—the peaks at 6.53°, 7.21°, and 9.19° corresponded to (002), (021) and (022) planes of MA_2_Pb_3_I_8_(DMSO)_2_. It is noteworthy that different preferential crystal orientations of intermediate phase were manipulated by adjusting the concentration of DMF. In addition, the perovskite phase was observed in 0DMF intermediate film, because the excessive water of solution owing to the strong hydration of DMSO provides greater bulk mobility to the MA and FA retained in the film, which accelerated the reaction with PbI_2_ to form perovskite phase during the spin-coating process [24]. By adding a small amount of DMF, the perovskite phase disappeared due to the reduced water of the solution, which has been identified by FTIR measurement. To further verify the contents of the intermediate phase, Attenuated Total Reflectance Fourier Transform Infrared (ATR-FTIR) spectra of intermediate films were measured. As shown in Figure 3c, vibrational bands observed at 3188 cm^−1^, which can be assigned to NH_3_^+^ in MAI(CH_3_NH_3_I) of intermediate phases [25]. A characteristic peak at 1714 cm^−1^ corresponded to stretching vibration of C=N in FAI[CH(NH_2_)_2_I]. The ATR-FTIR spectra of intermediate film based the hybrid FAMA cation and pure MA cation with 1DMF precursor solution is showed in Figure S1. The strong band at 1637 cm^−1^ is assigned to C=O stretching vibration of DMF molecule and 1018 cm^−1^ is assigned to S=O stretching vibration of DMSO molecule [26,27]. Thus, the composition of 0DMF intermediate film was a mixture of nonstoichiometric (FA_0.2_MA_1.8_)Pb_3_X_8_(DMSO)_2_ and perovskite phase. Additionally, the other films (1DMF, 2DMF, and 3DMF) are pure nonstoichiometric (FA_0.2_MA_1.8_)Pb_3_X_8_(DMSO·DMF). The formation of intermediate phase can be explained as the reaction between Lewis acids and bases, where lone pair electrons on oxygen in DMSO and DMF donate to Lewis acid Pb^2+^ in PbI_2_ to form adducts through Van der Waals interactions. Iodide (I^−^) in MAI and FAI are also strong donors and thereby readily forms an adduct with PbI_2_ [16,28,29]. Figure 4 shows the schematic crystal structure and composition of the intermediate phases (left), and then the perovskite phase (right) was formed due to the vaporization of solvent from the surface of the intermediate film after annealing.

**Figure 5:**
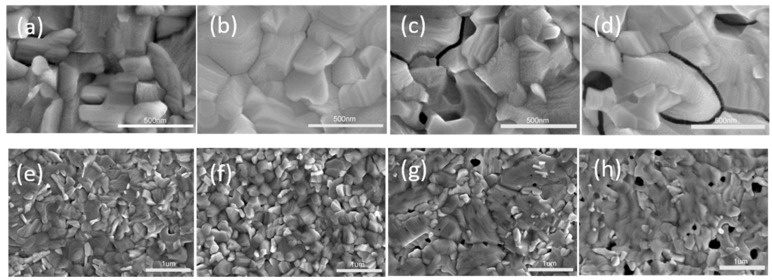
The SEM images of perovskite film by different precursors of 0DMF (**a**,**e**); 1DMF (**b**,**f**); 2DMF (**c**,**g**); 3DMF (**d**,**h**).

To further ascertain exactly how intermediate phase affects the surface morphology, crystallinity, and charge-carrier transport properties of the perovskite film, the annealed film was tested systematically. Figure 5 shows the scanning electron microscopy (SEM) images of perovskite films after annealing intermediate films. In the 0DMF case (Figure 5a,e), many grain boundaries are observed and randomly distributed in the film owing to the presence of perovskite phase and (FA_0.2_MA_1.8_)Pb_3_X_8_(DMSO)_2_ phase in intermediate films. The intermediate phase and perovskite phase have different growth directions that results in structural mismatch and is apt to produce more horizontal boundaries [19]. By adding a small amount of DMF (Figure 5b,f), the film was uniform, smooth, and showed almost no pinholes thanks to the pure intermediate phase before annealing. On further increasing the amount of DMF (Figure 5c,d,g,h), many pinholes appeared on the perovskite films although pure intermediate phase was also obtained in unannealed film. The rough film surface can be explained by the excess DMF evaporating more rapidly than DMSO during the annealing process [30], owing to the lower boiling point and weaker coordination ability with PbI_2_ [31]. This results revealed that the key of forming high quality perovskite film in air condition is following two aspects: Promoting the formation of the pure intermediate phase (FA_0.2_MA_1.8_)Pb_3_X_8_(DMSO·DMF) without the perovskite phase, and controlling the rate of solvent evaporation.

**Figure 6:**
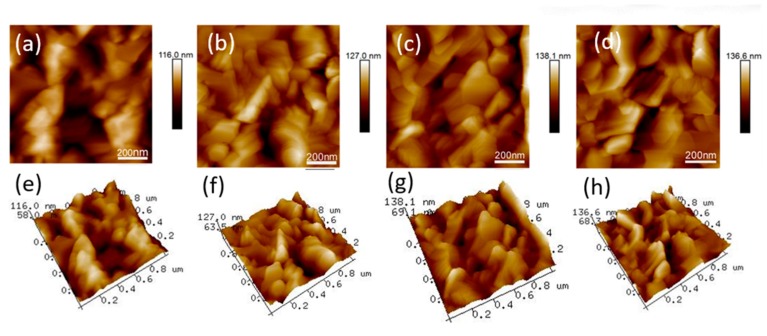
Atomic force microscopy (AFM) images of the perovskites film by different precursors (**a**,**e**) 0DMF, (**b**,**f**) 1DMF, (**c**,**g**) 2DMF, (**d**,**h**) 3DMF.

Figure 6 shows the atomic force microscopy (AFM) images of perovskite film. The root mean roughness values (Rq) were measured as 17.9, 16.3, 18.5, and 19 nm for film surface prepared by precursors of 0DMF, 1DMF, 2DMF, and 3DMF, respectively. The Rq of 1DMF sample was the smallest, which indicated a smooth surface of perovskite film. The corresponding 3D surface plot images (Figure 6e–f) apparently show the change of the surface roughness of perovskite films, which were in accordance with SEM images.

**Figure 7:**
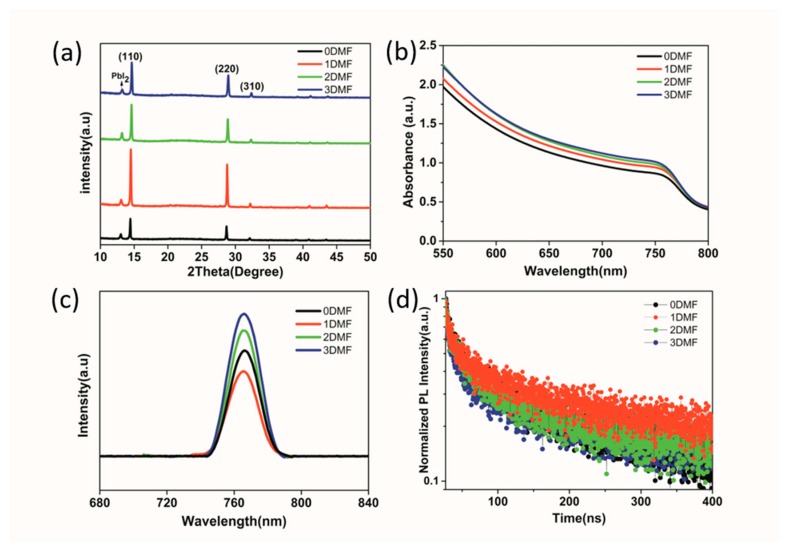
(**a**) XRD spectra of perovskite films on quartz by using different precursors; (**b**) UV−vis absorption spectra of perovskite films; (**c**) Steady-state photoluminescence (PL) spectra of different perovskite films on FTO/TiO2 (**d**) Normalized time-resolved PL (TRPL) spectra of the different perovskite films on glass.

Figure 7a shows XRD patterns of the perovskite film on quartz substrate. The intermediate phase was not observed in the XRD patterns of all annealed films. And three diffraction peaks at 14.28°, 28.56°, and 31.99° correspond to the (110), (220), and (310) planes of perovskite, respectively, confirming the entire intermediate phase was transformed into the perovskite phase [32]. In addition, the diffraction peak at 13.04° is PbI_2_ phase. It was reported that residual PbI_2_ could passivate the defects and reduce recombination in the perovskite film, and further improve the device performance [33]. It is apparent that the 1DMF diffraction peak of (110) and (220) planes were obviously higher than other samples—this could indicate higher crystallization of perovskite film [34,35]. Therefore, the results clearly demonstrate that the perovskite crystal quality was controlled by composition of the intermediate film. Figure 7b compares the UV−vis absorption spectra of the perovskite films on quartz substrate. The absorption edge of the four samples are the same. The 0DMF film exhibits less absorbance intensity, with increases the DMF amount, the intensity of absorption augment observably, which is attributed to the high quality of perovskite film [36]. Surprisingly, absorption of the 2DMF and 3DMF samples are higher than 1DMF samples, the small increase in absorption could be explained the scattering effect of the rough surface due to large pinholes in the films.

The charge transport properties and charge recombination dynamics of perovskite films were further studied by steady-state photoluminescence (PL) and time-resolved PL (TRPL). Figure 7c shows room temperature PL spectra of perovskite films on FTO/TiO_2_. The PL intensity of 1DMF film at 770 nm was weaker than the other films. That suggests the 1DMF film was better in charge transport and collection at the perovskite/TiO_2_ interface. Figure 7d shows normalized TRPL spectra of the perovskite films on glass. The TRPL decay curves could be fitted to a bi-exponential rate law:

I(t) = A_1_exp(−t/τ_1_) + A_2_exp (−t/τ_2_) (1)

where τ_1_ is faster decay that determines carriers extracted through the glass/perovskite interface, and τ_2_ is slower decay which represents the lifetime of free charge carriers [37]. The 1DMF film exhibited the largest PL lifetime with τ_2_ of 346 ns, that suggesting non-radiative recombination was effectively suppressed. These results indicated that the 1DMF films contained fewer traps and defects due to high-quality perovskite films [38,39,40].

**Figure 8:**
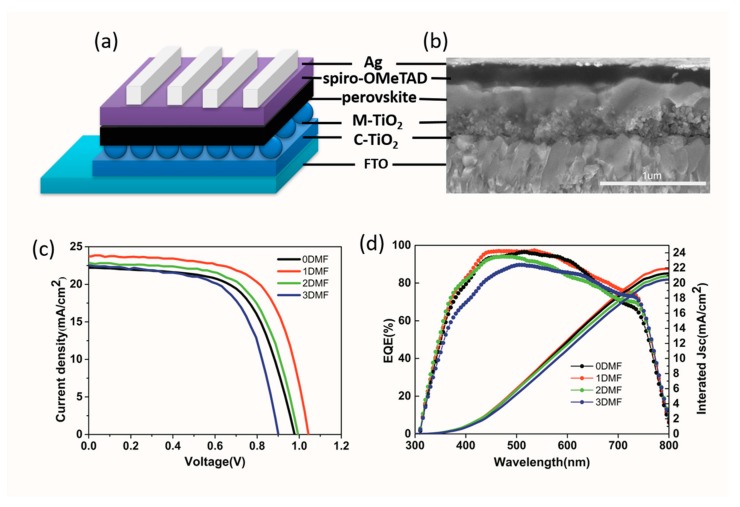
(**a**) Schematic device structure. (**b**) Cross-sectional SEM image of Ag/Spiro-MeOTAD/Perovskite/M-TiO_2_/C-TiO_2_/FTO device; (**c**) J−V curves of champion devices based four precursors; (**d**) the corresponding EQE curves.

**Figure 9:**
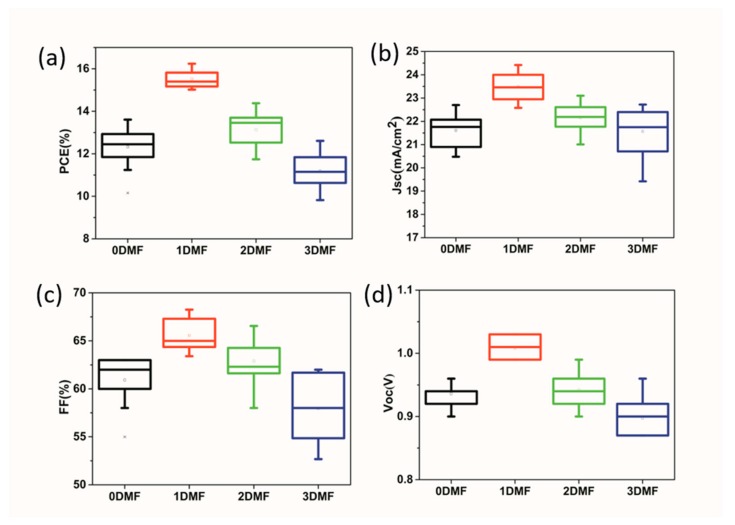
Photovoltaic statistics for the PSCs by four precursors. (**a**) PCE; (**b**) Jsc; (**c**) FF, and (**d**) Voc.

To assess the impact of different precursors on device performance, the mesoporous PSCs based on the structure of FTO/C-TiO_2_/M-TiO_2_/Perovskite/Spiro-MeOTAD/Ag (Figure 8a) were constructed. Figure 8b is cross-sectional SEM image of the PSCs. Figure 8c shows the J-V curves for PSCs based on four champion devices, and the corresponding photovoltaic parameters are listed in Table 1. The device using pure DMSO precursors solution exhibited a PCE of 13.61%, the open-circuit voltage (Voc) of 0.96 V, the short-circuit photocurrent density (Jsc) of 22.7 mA/cm^2^, and fill factor (FF) of 62.5%. The best performance was obtained by a 1DMF device (a Voc = 1.03 V, Jsc = 23.7 mA/cm^2^, FF = 66.5%, and PCE = 16.24%). The performance dropped for a 2DMF device (PCE of 14.38%), and a 3DMF device (12.61%). The obviously poor performance of 0DMF device was on account of the low crystallinity of perovskite film and many horizontal boundaries on the film surface. The excellent performance of 1DMF device could be attributed to the enhanced crystallinity and surface morphology of peroverskite film and better interface contact, which restrained electron-hole recombination and promoted charge transport at interface. For 2DMF and 3DMF devices, the main cause of poor performance was pinholes in the film which lead to electrons in the TiO_2_ recombine with holes on spiro-OMeTAD [41]. External quantum efficiency (EQE) further confirmed the J_SC_ results with the integrated Jsc of 22.05 mA/cm^2^ of a champion 1DMF device, as displayed in Figure 8d. Figure 9 shows photovoltaic statistics for the PSCs by four precursors. The 1DMF devices exhibited photovoltaic parameters with little standard deviation, leading to average Voc of 1.01 ± 0.02 V, Jsc of 23.49 ± 0.61 mA/cm^2^, FF of 65.6 ±1.6%, and PCE of 15.52 ± 0.72%. The correlative data are showed in Table S1.

**Figure 10:**
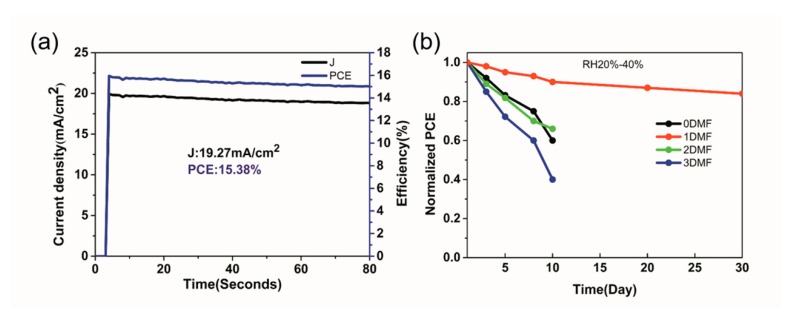
(**a**) Steady-state current density and PCE of 1DMF device under continuous illumination at maximum power point; (**b**) stability test for 0DMF, 1DMF, 2DMF, and 3DMF PSCs in air condition (RH 20–40%) without sealing.

Figure 10a shows the stable output of current density and PCE of a 1DMF device under continuous illumination at a bias voltage of 0.8 V. The 1DMF device works with an average PCE of 15.38%. The long-term stability of the devices was tested, which were stored in ambient air (RH20–40%) without sealing, as shown in Figure 10b. The 1DMF device exhibited the best stability among all the devices, with almost 86% of its initial PCE preserved after 30 days in air conditions which benefited from high-quality perovskite film. The surprised stability of the champion device in ambient air is mainly from the following aspects. First of all, the high crystallinity of the perovskite film deduced the probability of defect driven degradation and provided a basis for the device stability. In addition, the uniform film and compact interface prevented moisture into perovskite film, which further enhanced the humidity stability of device. Finally, the unencapsulated device was stored in dark air condition, which reduced the damage from light [42,43,44,45].

**Figure 11:**
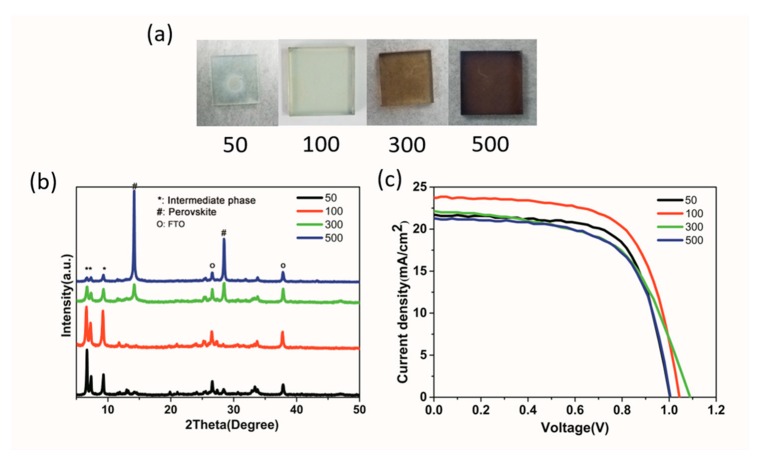
(**a**) The intermediate film photo by different quantity of EA (50 μL, 100 μL, 300 μL, 500 μL); (**b**) XRD spectra of intermediate films; (**c**) J-V curves of devices.

To further examine the effect of the anti-solvent quantity to form intermediate film, Figure 11a shows the intermediate film photo of different ethyl acetate (EA) quantity (50 μL,100 μL, 300 μL, 500 μL) with 1DMF precursor solution. The intermediate film color changes from colorless to light brown and to deep brown with the increase of EA quality, indicating the perovskite phase component in intermediate film increased, which has been confirmed by XRD patterns (Figure 11b). This transformation is explained by the excess solvent washed by sufficient EA that resulted in fast crystallization of perovskite phases during the spin-coating process. Figure 11c shows the typical J-V curves of PSCs by dripping different quantity EA; the photovoltaic parameters are listed in Table 2. The corresponding photovoltaic statistics of the devices are shown in Table S2. The same volume (100 μL) CB and methyl acetate (MA) were also used as anti-solvent in this work; the J-V curves and photovoltaic data are shown in Figure S2 and Table S3. It can be seen that both CB and MA devices show lower performance than the EA device, because the hydration of CB was poor and that of MA was too strong, while EA had proper hydration under this condition (RH 20–30%).

**Figure 12:**
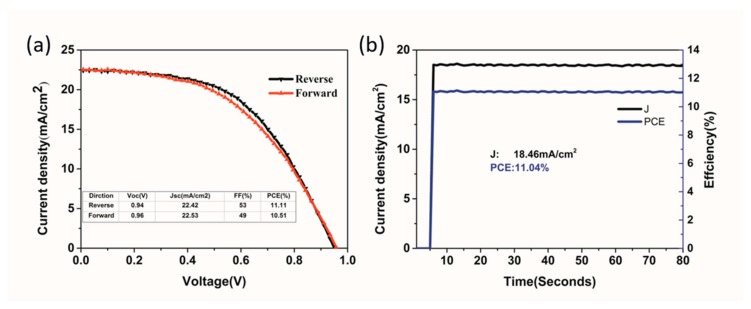
(**a**) Reverse and forward J-V curves of large area (100 mm^2^) device, and inserted table is the corresponding photovoltaic parameters under forward and reverse scans; (**b**) steady-state current density and efficiency of 100 mm^2^ device under continuous illumination at maximum power point (0.6V).

Finally, we fabricated a larger device with an active area of 100 mm^2^ in the same air condition; the J-V curves were shown in Figure 12a. The PCE of a device is 11.11% with negligible hysteresis when measured from reversing to forwarding. The inserted table is the corresponding photovoltaic parameters under forward and reverse scans, with the average Voc of 0.95 V, Jsc of 22.475 mA/cm^2^, FF of 51%, and PCE of 10.81%. Figure 12b shows a stable output of current density and PCE of 100 mm^2^ device under one sun continuous illumination at maximum power point (0.6V). The PSCs worked with the PCE of 11.04%, which indicated good stability of power output.

## 4. Conclusions

In conclusion, we evaluated the effects of precursor solution, anti-solvent, and moisture on the formation of intermediate phase, and the mechanism was discussed about PSCs in air condition (RH 20–30%). The excessive water of solution owing to the strong hydration of DMSO promotes the formation of perovskite phase in intermediate film, that results in poor quality perovskite film after annealing. The pure intermediate phase (FA_0.2_MA_1.8_)Pb_3_X_8_(DMSO·DMF) and appropriate rate of solvent evaporation are key to forming high-quality perovskite layer in air condition. In addition, 100uL of EA was appropriate quantity to obtain pure intermediate phase in this work. Eventually, the best device PCE of 16.24% was obtained owing to high-quality perovskite film, compact interface contacts, and excellent charge transport properties. Moreover, the long-term stability of device was impressive, retaining 86% of its original performance after 30 days in air condition (RH 20–40%) without sealing. At the same humidity condition, the device of 100 mm^2^ active area was fabricated, with PCE of 11.11% with a small hysteresis. This work contributes to further understanding the function of solvent engineering and film-forming mechanism from intermediate phase to perovskite phase, and to give an orientation for PSCs production at the commercial level.

## Figures and Tables

**Table 1:** Photovoltaic parameters of champion devices based on different precursor.

Precursor	Voc (V)	Jsc (mA/cm^2^)	FF (%)	PCE (%)
0DMF	0.96	22.7	62.5	13.61
1DMF	1.03	23.7	66.5	16.24
2DMF	0.99	22.8	63.7	14.38
3DMF	0.90	22.5	62.3	12.61

**Table 2:** Photovoltaic parameters of device by using different quantity of anti-solvent EA.

Anti-Solvent Volume (μL)	Voc (V)	Jsc (mA/cm^2^)	FF (%)	PCE (%)
50	0.98	21.68	71.0	14.97
100	1.03	23.70	66.5	16.24
300	1.08	22.11	58.3	13.93
500	1.01	21.26	64.3	13.81

## Supplementary Materials

The following are available online at https://www.mdpi.com/2079-4991/9/7/915/s1. Figure S1: The ATR-FTIR spectra of hybrid FAMA cation and pure MA cation intermediate films, Table S1: The photovoltaic parameters of device fabraication by four precursors (0DMF,1DMF,2DMF and 3DMF), Table S2: The photovoltaic statistics of device by using different quantity of EA. Figure S2: The J-V curves of device by dripping chlorobenzene (CB) and methyl acetate (MA), Table S3: corresponding photovoltaic parameters.
