# Supplementary material for: Solvent Engineering for Intermediates Phase, All-Ambient-Air-Processed in Organic–Inorganic Hybrid Perovskite Solar Cells
Source: Nanomaterials (Basel). 2019 Jun 26;9(7):915. doi: 10.3390/nano9070915 (PMC6669498; doi:10.3390/nano9070915)
Supplement: Supplementary file 1 [file nanomaterials-09-00915-s001.pdf]

Supplementary Material

Solvent Engineering for Intermediates Phase, All-Ambient-Air-Processed in Organic–Inorganic Hybrid Perovskite Solar Cells

Lei Shi, Huiying Hao *, Jingjing Dong *, Tingting Zhong, Chen Zhang, Jiabin Hao, Jie Xing and Hao Liu

School of Science, China University of Geosciences, Beijing 100083, China; 2019170012@cugb.edu.cn (L.S.); 2019180018@cugb.edu.cn (T.Z.); 2019180017@cugb.edu.cn (C.Z.); jiajooy@163.com (J.H.); xingjie@cugb.edu.cn (J.X.); 2014010007@cugb.edu.cn (H.L.)

* Correspondence: huiyinghaoL@cugb.edu.cn (H.H.); jjdong@cugb.edu.cn (J.D.)

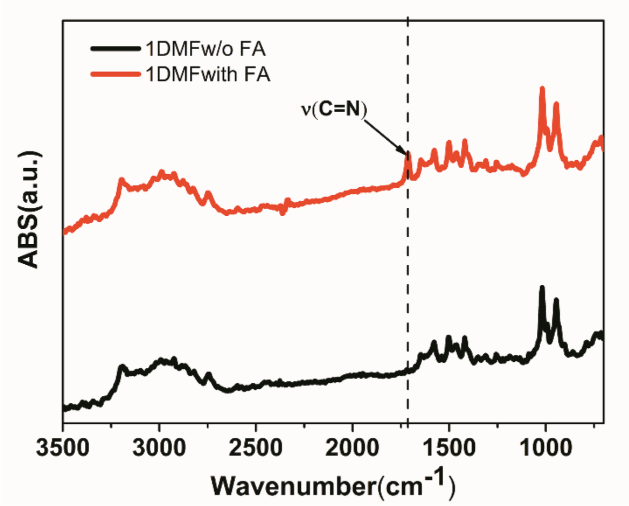

Figure S1. The ATR-FTIR spectra of hybrid FAMA cation and pure MA cation intermediate films with 1DMF precursor solution.

Table S1. The photovoltaic parameters of device by using different precursors.

Precursor	Voc(V)	Jsc(mA/cm2)	FF	PCE (%)
0DMF	0.94	21.76	0.61	12.42
	0.94	20.60	0.58	11.24
	0.94	22.62	0.59	12.49
	0.92	21.55	0.63	12.45
	0.92	20.90	0.60	11.55
	0.94	22.29	0.63	13.10
	0.92	21.74	0.60	12.04
	0.94	21.97	0.63	12.93
	0.94	22.07	0.62	12.85
	0.90	20.48	0.55	10.16
	0.92	20.90	0.62	11.85
	0.96	22.07	0.63	13.40
	0.94	20.69	0.63	12.27

	0.96	22.70	0.62	13.61
	0.96	21.86	0.60	12.53
1DMF	1.03	23.70	0.67	16.24
	0.99	24.38	0.63	15.32
	1.03	24.40	0.65	16.22
	1.03	22.86	0.67	15.86
	1.01	23.11	0.66	15.33
	1.03	22.58	0.65	15.11
	1.00	23.45	0.65	15.17
	0.99	24.42	0.64	15.56
	1.01	23.66	0.64	15.35
	1.01	23.65	0.65	15.64
	0.99	23.46	0.65	15.02
	1.01	22.84	0.68	15.65
	0.99	24.00	0.64	15.14
	0.99	22.95	0.68	15.40
	1.01	22.95	0.68	15.82
2DMF	0.92	23.10	0.64	13.55
	0.92	21.37	0.64	12.53
	0.96	21.85	0.61	12.89
	0.92	21.34	0.60	11.78
	0.92	22.19	0.62	12.71
	0.99	22.81	0.62	14.07
	0.90	22.61	0.58	11.74
	0.99	22.18	0.62	13.68
	0.94	21.01	0.62	12.17
	0.92	22.19	0.62	12.71
	0.99	22.81	0.64	14.38
	0.94	22.28	0.64	13.46
	0.94	21.77	0.67	13.62
	0.94	22.28	0.65	13.70
	0.94	22.49	0.66	13.94
3DMF	0.87	22.18	0.62	11.96
	0.87	21.23	0.62	11.52
	0.92	22.27	0.57	11.75
	0.87	22.40	0.56	10.84
	0.90	20.71	0.58	10.75
	0.90	22.29	0.59	11.84
	0.90	22.50	0.62	12.61
	0.87	19.96	0.61	10.67
	0.90	22.71	0.59	12.03
	0.96	19.42	0.53	9.82
	0.90	22.72	0.55	11.15
	0.92	21.55	0.54	10.63
	0.94	20.16	0.62	11.69
	0.87	21.65	0.54	10.25
	0.87	21.75	0.55	10.38

Table S2. The photovoltaic statistics of device by using different quantity of anti-solvent.

Anti-solvent volume (uL)	Voc (V)	Jsc(mA/cm^2)	FF	PCE (%)
50	0.99	21.98	0.64	13.95
	0.96	18.74	0.66	11.87
	0.96	21.87	0.56	11.71
	0.96	20.40	0.66	12.83
	1.01	21.57	0.68	14.74
	1.01	21.68	0.67	14.56
	0.98	21.46	0.71	14.89
	0.98	21.68	0.71	14.97
	0.96	22.95	0.68	14.90
	0.99	20.84	0.69	14.13
	0.99	22.74	0.64	14.34
	1.02	20.05	0.60	12.34
	0.99	20.39	0.60	12.14
300	1.02	23.37	0.54	12.78
	1.05	23.48	0.52	12.91
	1.03	23.37	0.54	13.01
	1.08	21.89	0.58	13.63
	1.08	22.00	0.59	13.74
	1.04	22.11	0.52	11.85
	1.08	22.11	0.58	13.93
	1.00	21.47	0.62	13.25
	1.01	21.57	0.63	13.63
	1.03	23.47	0.51	12.34
	0.99	21.97	0.59	12.88
	1.05	23.37	0.53	12.90
	1.00	20.06	0.62	12.41
500	1.01	21.26	0.64	13.81
	0.92	21.15	0.64	12.52
	0.94	21.68	0.64	13.01
	0.94	22.11	0.61	12.71
	0.92	21.26	0.61	11.91
	0.96	20.62	0.61	12.71
	0.94	21.58	0.66	13.45
	0.92	20.94	0.59	11.45
	0.96	21.15	0.64	13.06
	0.90	20.73	0.59	10.99
	0.94	20.52	0.60	11.59
	0.94	17.45	0.58	9.54
	0.94	19.25	0.581	10.51

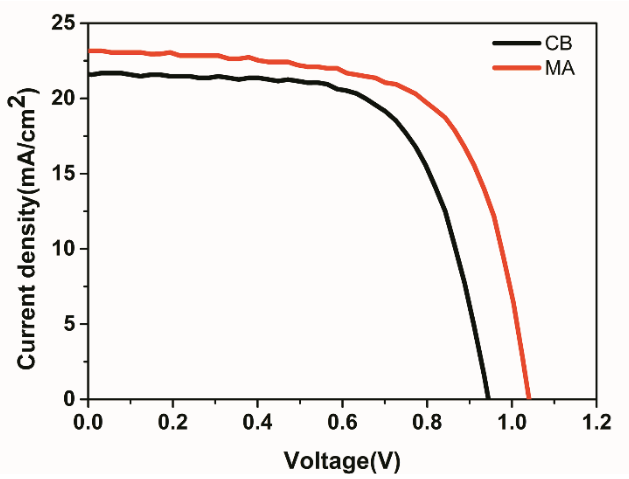

Figure S2. J−V curves of devices based different anti-solvent (CB and MA).

Table S3. The photovoltaic parameters of device based different anti-solventv (MA and CB).

Anti-solvent	Voc(V)	Jsc(mA/cm2)	FF(%)	PCE(%)
CB	0.94	21.58	66.3	13.45
MA	1.03	23.16	66.2	15.78
